# Association Between Preoperative Short Physical Performance Battery and Hospitalization-Associated Disability After Transcatheter Aortic Valve Implantation

**DOI:** 10.7759/cureus.89977

**Published:** 2025-08-13

**Authors:** Yutaro Ohnishi, Tsubasa Yokote, Kenta Kawamitsu, Taichi Ogami, Takatoshi Nishimura, Takayuki Uchida, Shoji Kawakami

**Affiliations:** 1 Rehabilitation, Iizuka Hospital, Iizuka, JPN; 2 Cardiovascular Surgery, Iizuka Hospital, Iizuka, JPN; 3 Cardiology, Iizuka Hospital, Iizuka, JPN

**Keywords:** cardiac rehabilitation (cr), critical aortic stenosis, hospitalization-associated disability, short physical performance battery, transcatheter aortic valve implantation

## Abstract

Background

The Short Physical Performance Battery (SPPB) test can easily evaluate lower limb function, and its reliability in patients with heart disease has been verified. This study aimed to examine whether the preoperative SPPB, a lower extremity frailty test, predicts hospitalization-associated disability (HAD), a complication linked to adverse long-term outcomes, after transcatheter aortic valve implantation (TAVI).

Methodology

We retrospectively studied 92 consecutive TAVI patients (median age = 85 years, 63% female) treated between 2019 and 2024. Pre-TAVI SPPB was administered within three days before the procedure and divided into frail (<9) and robust (≥9) groups. All patients received individualized rehabilitation (low‑ to moderate‑intensity walking or cycle ergometer, resistance training targeting major lower limb muscle groups, and functional activities of daily living practice; 40-60 minutes, ≥5 days/week) beginning postoperative day one to two. Propensity scores were calculated with age, sex, Charlson Comorbidity Index >2, postoperative ambulation day, estimated glomerular filtration rate, and Geriatric Nutritional Risk Index, and balanced by overlap weighting. Logistic regression (overlap-weighted only) tested the association between the SPPB group and HAD, defined as a ≥5-point decline in the Barthel Index from the preoperative baseline to discharge.

Results

HAD occurred in 26.1% of the study. Adjusted analysis showed no significant association between preoperative frailty status and HAD (adjusted odds ratio = 1.11, 95% confidence interval = 0.41-2.98, p = 0.83).

Conclusions

An SPPB score <9 did not independently predict HAD after TAVI. These findings suggest that SPPB alone may be insufficient for preoperative HAD risk stratification, and that a multidimensional assessment, potentially including baseline ADL capacity and postoperative activity monitoring, remains necessary to identify high-risk patients.

## Introduction

The incidence of aortic stenosis has increased in recent years, mainly due to progressive aortic valve calcification in aging populations [1]. Transcatheter aortic valve implantation (TAVI) is considered the treatment of choice for aortic stenosis in older patients because it is minimally invasive. Nevertheless, preoperative frailty is linked to worse activities of daily living (ADLs) [2] at discharge and reduced long-term survival [3]. Hospitalization-associated disability (HAD), defined as a new loss of basic ADL capacity that develops during the index stay, has recently emerged as a particularly important in-hospital complication. Unlike delirium or early mortality, HAD directly reflects functional decline and often triggers downstream consequences such as prolonged institutionalization, loss of independence, and greater long-term healthcare use, and higher care-level needs [4-6]. Therefore, preventing HAD is a clinical priority after TAVI. Early individualized rehabilitation begun immediately after cardiac procedures has been shown to mitigate HAD risk in older adults [7]. It is important to evaluate before TAVI to know the risk of needing intensive rehabilitation at an early stage. Factors that have been reported to affect HAD in TAVI patients include age, body mass index (BMI), chronic renal failure, and other factors that affect frailty [7]. Several frailty assessments have been validated before TAVI, including gait speed, grip strength, the Katz Index, the six-minute walk test, and the Japan-Cardiovascular Health Study (J-CHS) criteria [8-10]. Another well-validated, lower extremity-focused frailty measure is the Short Physical Performance Battery (SPPB) [11-13]. The SPPB is a bedside composite test of balance, usual gait speed, and the five times sit-to-stand; it can be completed in ≤5 minutes with only a stopwatch and a standard chair [14]. This simplicity imposes minimal burden on patients and staff, making the tool feasible even for frail or clinically unstable individuals. It has also been tested for reliability and validity in patients with cardiac disease [15], and even in patients who have undergone TAVI, frailty assessed using the SPPB preoperatively has been shown to predict postoperative delirium [16] and mortality [17].

However, to our knowledge, no study has examined the association between pre-TAVI SPPB scores and HAD. SPPB is associated with post-TAVI prognosis, but its relationship with HAD, which is a risk factor for long-term prognosis, is unclear. Therefore, we investigated whether a lower pre-TAVI SPPB score predicts HAD. We hypothesize that patients who are found to be frail on the SPPB are at high risk for HAD, and that early detection will lead to targeted rehabilitation strategies to maintain function.

## Materials and methods

Patients

This study included patients with aortic stenosis who underwent TAVI at the Aso Iizuka Hospital (Fukuoka, Japan) between February 2019 and February 2024. At that facility, rehabilitation is prescribed for all TAVI patients. Patients who died during hospitalization, developed severe stroke during the postoperative hospitalization period, cases in which treatment of an existing disease was initiated with continued hospitalization, or had missing SPPB data (patients for whom SPPB could not be attempted due to risk management) were excluded from the study.

Ethical considerations

The study was conducted in accordance with the Declaration of Helsinki and with due consideration for the protection of the study participants. Approval was obtained from the Ethics Committee of Iizuka Hospital (Fukuoka, Japan) (approval number: R21135). Following approval by the Ethics Committee, informed consent was posted on the hospital website using the opt-out method.

Study design

For this retrospective, observational study, data were obtained from the patients’ medical records. These encompassed age, sex, BMI, dementia diagnosis, length of hospital stay, postoperative length of hospital stay, Charlson Comorbidity Index (CCI) [18] at admission, postoperative cerebrovascular and respiratory complications, complete atrioventricular block (pacemaker implanted), results for preoperative blood tests (estimated glomerular filtration rate (eGFR), serum albumin levels), echocardiographic findings (left ventricular ejection fraction), Geriatric Nutritional Risk Index (GNRI; calculated as 14.89 × serum albumin (g/dL) + 41.7 × (current weight/ideal weight), number of days to start ambulation postoperatively, rehabilitation intervention rate (days of rehabilitation intervention/postoperative hospital stay calculated as ×100 (%)), SPPB scores, and Barthel Index (BI) before and after surgery. This study uses complete data without missing data.

Definition of frailty based on SPPB scores

The SPPB, which can comprehensively and objectively assess physical function, was used as an index to evaluate frailty before TAVI [16,17]. The SPPB assesses the following three categories: standing balance, usual gait speed, and five times sit-to-stand tests, with a total score ranging from 0 to 12 points (0 to 4 points per category) [14]. Using cutoffs from previous studies with risk of ADL impairment, institutionalization, and death as outcomes, patients scoring 9 or higher were included in the robust group and patients scoring less than 9 were included in the frail group [14,19].

Definition of HAD

HAD was assessed using BI. The BI assesses ADLs, such as eating, walking, and changing, and is measured on a 100-point scale comprising 5-15 points for each of the 10 items, with higher scores indicating greater independence [20]. The BI was assessed on the day of admission and the day before discharge, respectively. The incidence of HAD was defined as a decrease in BI by ≥5 points at discharge from BI before TAVI [21].

Rehabilitation after TAVI

Rehabilitation is provided to all TAVI patients at the hospital. It begins the day after surgery and includes ambulation, aerobic exercises, and ADL exercises until the day before discharge.

Statistical analysis

Patient characteristics, HAD, and physical function were compared between the robust and frail groups stratified according to SPPB scores. Data are shown as median (interquartile range), regardless of normality. Comparisons between the two groups were analyzed using the Wilcoxon rank sum test for continuous variables and Fisher’s exact test for categorical variables. The propensity score method was employed to balance confounding factors and reduce bias because direct adjustment for confounding factors in multivariate analyses can result in unstable estimates when sample sizes are small. The propensity score method effectively adjusts for confounding factors in observational studies by creating comparable groups, balancing covariates, and improving the reliability of treatment effect estimates [22]. Propensity scores were estimated with a logistic‐regression model that included age [23,24], sex [23,24], CCI [25], postoperative day of first ambulation [26], eGFR [11], and nutritional status assessed by the GNRI [11], variables previously linked to frailty, postoperative functional decline, and hospital‑acquired disability. The scores were adjusted using overlap weighting [27], which is used to calculate the average treatment effect of an overlapping population. We selected overlap weighting because it retains the full sample, down‑weights units with extreme propensity scores, and achieves superior covariate balance and therefore greater statistical efficiency than matching or inverse probability weighting in small observational cohorts [27,28]. Overlapping populations have similar covariate distributions, ensuring clinical equality and eligibility for similar trials [27,28]. By applying overlap weighting to the two groups, the distribution of confounding factors was equalized between the groups, and the absolute standardized mean differences were ≤0.10 [27,28]. HAD was compared between the two groups after overlap weighting. Statistical analyses were performed using STATA (Statanow, version 18.5) and R software (R, version 4.2.2), with the significance level set at 5%. We used both Stata and R to enhance transparency and reproducibility. Stata handled data management and descriptive statistics, while R performed the primary analyses, including propensity score overlap weighting, sensitivity analyses, and figure generation. All analyses were conducted under the supervision of statistical experts.

## Results

Patients

**Figure 1:**
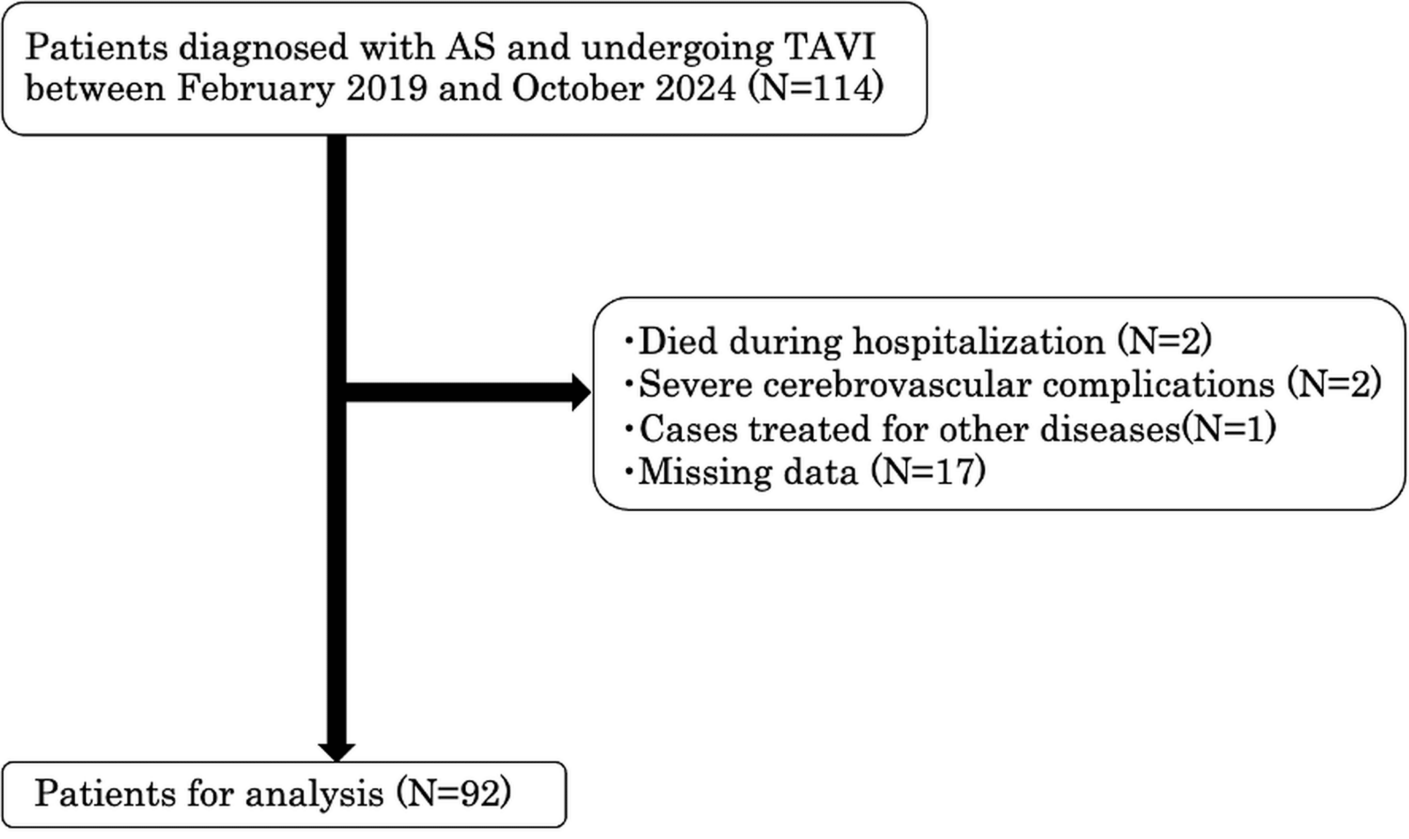
Flowchart of the patient selection process. AS: aortic stenosis; TAVI: transcatheter aortic valve implantation

This study included 114 patients; among them, 22 were excluded (death: 2; severe complications (developed severe stroke during the postoperative hospitalization period): 2; started treatment for another disease: 1; and cases with assessment deficits, including risk management: 17), leaving 92 patients for the final analysis (Figure 1). The median patient age was 85 years (quartiles = 82 and 88 years), and 58 (63.0%) patients were female. The incidence of HAD was 26.1%.

Baseline characteristics of the two SPPB groups

Table 1 presents the baseline comparisons between the two groups stratified by the SPPB scores. The robust and frail groups included 51 (55.4%) and 41 (44.6%) patients, respectively. Significant differences in sex, length of hospital stay, postoperative length of hospital stay, rate of discharge to the original place of residence, preoperative BI, and rate of rehabilitation intervention (p < 0.05) were observed between groups. No significant difference was observed in the HAD between the two groups (12 (29.3%) vs. 12 (24.0%), p = 0.64).

**Table 1:** Comparison between the two groups stratified by the SPPB. Data are presented as median (Q1, Q3) or n (%). Comparisons between the two groups were analyzed using the Wilcoxon rank sum test for continuous variables and Fisher’s exact test for categorical variables. The significance level was defined as p < 0.05. BMI: body mass index; eGFR: estimated glomerular filtration rate; Alb: serum albumin; GNRI: Geriatric Nutritional Risk Index; CCI: Charlson Comorbidity Index; SPPB: Short Physical Performance Battery; BI: Barthel Index; HAD: hospitalization-associated disability

	Short Physical Performance Battery				
Characteristic	Overall, N = 92	Frailty (SPPB <9), N = 41 (44.6%)	Robust (SPPB ≧9), N = 51 (55.4%)	Statistics	P-value
Age, years	85.0 (82.0, 88.0)	86.0 (84.0, 88.0)	85.0 (82.0, 88.0)	0.721	0.47
Female, sex	58.0 (63.0%)	33.0 (80.5%)	25.0 (49.0%)	-	0.002
Surgical technique, transfemoral approach	90.0 (97.8%)	40.0 (97.6%)	50.0 (98.0%)	-	1
BMI, kg/m^2^	22.8 (20.5, 25.5)	21.9 (20.4, 24.2)	23.1 (20.7, 26.0)	-1.002	0.32
Length of hospital stay, days	16.5 (12.0, 27.5)	21.0 (14.0, 29.0)	15.0 (11.0, 25.0)	2.254	0.024
Postoperative length of hospital stay, days	12.0 (9.0, 18.5)	14.0 (10.0, 22.0)	11.0 (9.0, 15.0)	2.579	0.01
eGFR, mL/minute/1.73 m^2^	49.8 (39.4, 59.4)	49.3 (37.9, 58.6)	51.1 (40.5, 60.0)	-0.742	0.46
Alb, g/dL	3.6 (3.3, 4.0)	3.6 (3.3, 3.8)	3.6 (3.3, 4.0)	-0.565	0.57
GNRI	96.2 (90.6, 103.6)	96.4 (91.1, 101.7)	96.2 (89.4, 105.3)	-0.544	0.59
Left ventricular ejection fraction, %	64.0 (55.5, 67.0)	63.0 (53.0, 66.0)	64.0 (59.0, 67.0)	-1.452	0.15
CCI score >2	51.0 (55.4%)	25.0 (61.0%)	26.0 (51.0%)	-	0.4
Postoperative cerebrovascular complications, number of individuals	0.0 (0.0%)	0.0 (0.0%)	0.0 (0.0%)	-	NA
Postoperative complete atrioventricular block, number of individuals	10.0 (10.9%)	6.0 (14.6%)	4.0 (7.8%)	-	0.33
Postoperative respiratory complications, number of individuals	0.0 (0.0%)	0.0 (0.0%)	0.0 (0.0%)	-	NA
Discharge to original place of residence, number of individuals	84.0 (91.3%)	34.0 (82.9%)	50.0 (98.0%)	-	0.02
Dementia	8.0 (8.7%)	5.0 (12.2%)	3.0 (5.9%)	-	0.46
Preoperative SPPB total score, points	9.0 (6.0, 11.0)	6.0 (3.0, 7.0)	11.0 (10.0, 12.0)	-8.286	<0.001
Preoperative BI, points	100.0 (85.0, 100.0)	85.0 (70.0, 100.0)	100.0 (95.0, 100.0)	-4.888	<0.001
Initiation date of ambulation, days	2.0 (1.0, 2.0)	2.0 (1.0, 2.0)	2.0 (1.0, 2.0)	1.428	0.15
HAD, persons	24.0 (26.1%)	12.0 (29.3%)	12.0 (24.0%)	-	0.64
Rehabilitation intervention rate, %	87.5 (78.6, 92.3)	88.9 (82.9, 93.8)	85.7 (77.4, 90.0)	2.206	0.027

Standardized mean difference between factors in the propensity score method

**Figure 2:**
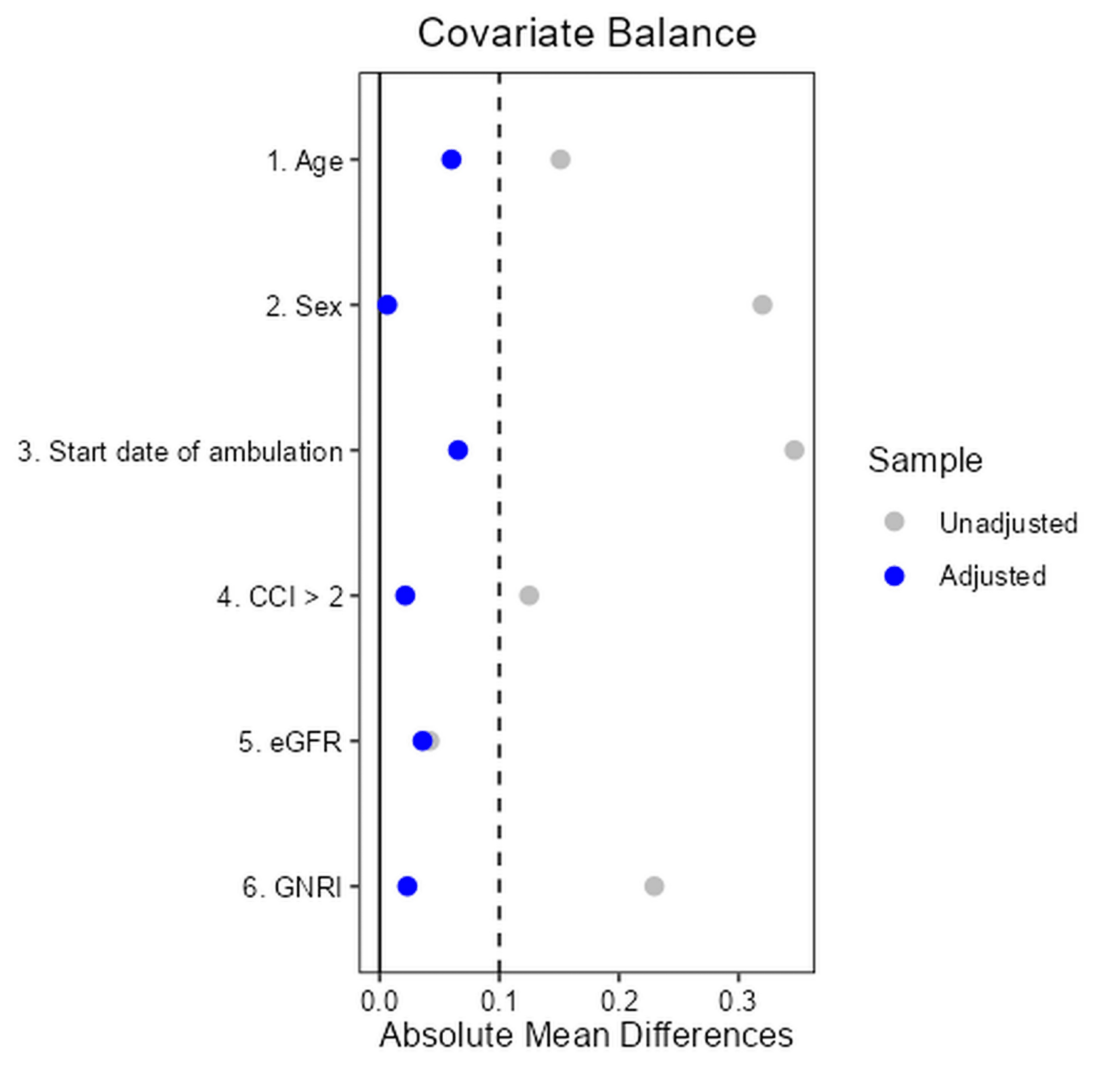
Standardized mean difference between factors after overlap weighting. Standardized mean differences (SMDs) in baseline covariates between the robust and frail groups are shown. SMDs ≤0.10 suggest adequate balance. OW: overlap weighting; CCI: Charlson Comorbidity Index; eGFR: estimated glomerular filtration rate; GNRI: Geriatric Nutritional Risk Index

Table 2 and Figure 2 show the standardized mean differences between the factors after overlapping weighting using propensity scores. After overlap weighting, all baseline covariates were balanced between groups; the absolute standardized mean differences for every variable were ≤0.10, a commonly accepted threshold indicating negligible imbalance in propensity score analyses [28].

**Table 2:** Baseline characteristics after overlap weighting using propensity scores. Data are presented as median (Q1, Q3) or n (%). SMD: standardized mean difference; CCI: Charlson Comorbidity Index; eGFR: estimated glomerular filtration rate; GNRI: Geriatric Nutritional Risk Index

Characteristics	Frailty (SPPB <9), N = 19	Robust (SPPB ≧9), N = 19	SMD after weighting
Age, years	85 (82, 88)	85 (81, 88)	-0.0600
Female, sex	13 (70%)	13 (70%)	-0.0063
Postoperative initiation date of ambulation	2 (1, 2)	2 (1, 2)	0.0655
CCI score >2	11 (59%)	11 (59%)	0.0214
eGFR	51 (41, 59)	49 (37, 61)	0.0358
GNRI	95 (88, 103)	97 (91, 103)	0.0231

Association between preoperative SPPB scores and HAD

After overlap weighting (Table 3), the effective sample size was 19 per group, and preoperative SPPB scores were not significantly associated with HAD (OR = 1.11; 95% CI = 0.41, 2.98, p = 0.83).

**Table 3:** Postoperative length of hospital stay and hospitalization-associated decline after overlap weighting. The significance level was defined as p < 0.05.

	OR	95% CI	P-value
Hospitalization-associated decline	1.11	0.41, 2.98	0.834

Sensitivity analyses reclassifying frailty using different cutoff values (SPPB <8 and SPPB <10) similarly found no significant association with HAD after overlap weighting (adjusted OR = 0.84 and 1.28, both p > 0.60) (Table 4).

**Table 4:** Sensitivity analysis with hospitalization-associated decline as outcome. The significance level was defined as p < 0.05. SPPB: Short Physical Performance Battery

Cut‑off definition	OR	95% CI	P-value
Primary analysis (SPPB)	1.11	0.41, 2.98	0.834
Sensitivity A (SPPB)	0.84	0.31, -2.32	0.741
Sensitivity B (SPPB)	1.28	0.48, -3.40	0.625

## Discussion

This study investigated the association between preoperative SPPB scores and HAD in patients who underwent TAVI. The SPPB pre-TAVI score did not affect HAD (p = 0.83).

In this study, the incidence of HAD in patients who underwent TAVI was 26.1%, which was comparable to that reported in a previous study (24.4%) [4]. No significant difference in the incidence of HAD was observed between the two groups stratified according to pre-TAVI SPPB scores. Sensitivity analyses re‑classifying frailty with alternative cut‑offs (SPPB <8 and SPPB <10) likewise showed no significant association with HAD after overlap weighting. This consistency across thresholds suggests that the composite SPPB score, regardless of the cut‑off chosen, contributes little to HAD prediction in this study. Honda et al. [29] investigated the factors affecting HAD in patients undergoing cardiac surgery and reported that the preoperative gait speed was influential. The SPPB includes gait speed, but its range is limited because it is a composite score. Additionally, the pre-TAVI scores may have been clustered higher, which could have resulted in a ceiling effect. Additionally, hospital-level care factors were not fully captured, which may have reduced their discriminatory power. Thus, differences in assessment methods may account for some of the varying findings. Because inactivity during hospitalization is a major driver of HAD, our rehabilitation protocol followed Japanese guidelines of low-to-moderate-intensity aerobic exercise ≥5 days/week and resistance training 2-3 days/week to preserve activity and ADLs [15]. Consistent with reports that proactive rehabilitation after TAVI facilitates ADL recovery [30], the frail group in our study experienced a longer postoperative stay and received greater rehabilitation exposure than the robust group. Although we did not quantify exercise dose, this greater exposure could have mitigated HAD occurrence among frail patients. At the same time, prolonged length of hospital stay in older TAVI patients is associated with long-term mortality [31] and may increase baseline risk. These opposing influences may have attenuated differences attributable to pre‑TAVI SPPB. These results suggest that a more intensive rehabilitation intervention compared to that in the robust group may have contributed to the prevention of HAD in the frail group. However, a detailed assessment of ADL before TAVI was not performed. The BI varies in the distribution of points and content difficulty for each item. Accordingly, a lower pre‑TAVI BI may not always denote greater vulnerability; in some patients, it could simply indicate more room for improvement, and the risk of HAD may be lower. In addition, maintaining a high level of postoperative physical activity is effective in preventing HAD [32]. In future studies, replacing the BI with more granular ADL measures such as the Functional Independence Measure may reduce scoring variability and capture subtler functional changes relevant to HAD. In addition, this study did not investigate the level of objective physical activity and exercise therapy after TAVI. Future studies should examine the influence of these factors on HAD incidence. In future prospective studies, the dose and content of postoperative rehabilitation should be quantified. Preoperative physical activity should be assessed using validated questionnaires and, where possible, device-based monitoring. Postoperative complications should also be specified in advance and graded according to severity using standardized definitions.

This study had certain limitations. First, the possibility of bias in patient background factors cannot be ruled out because of the small sample size and single-center nature of this study. We used propensity score adjustment and overlap weighting to improve covariate balance, but these procedures reduced the effective sample size; as a result, statistical power to detect modest associations was limited, as reflected by the wide confidence intervals. In addition, although overlap weighting is a causal inference-oriented method, residual confounding may persist; therefore, our estimates should be interpreted as associations, interpreted cautiously, and validated in larger multicenter cohorts. Second, the study used a retrospective design, and the data obtained may not have eliminated potential confounders. Furthermore, because patients who died or experienced severe stroke were excluded, survivor bias may have led to an underestimation of HAD incidence and limited the generalizability of our findings. Therefore, prospective follow-up studies are required to validate these findings.

## Conclusions

Pre‑TAVI SPPB scores were not associated with HAD. This suggests other preoperative determinants, including specific SPPB domains, require study. HAD risk likely reflects domain performance (balance, endurance), baseline ADLs, and postoperative activity. In the frailty group, greater rehabilitation and longer stay may have mitigated HAD. This remains a hypothesis because dose and activity were not quantified. Prospective studies should quantify rehabilitation dose/content and objective activity, as well as test domain‑specific and ADL‑based predictors.
